# Effect of Inoculum Microbial Diversity in Ex Situ Biomethanation of Hydrogen

**DOI:** 10.3390/bioengineering9110678

**Published:** 2022-11-10

**Authors:** Washington Logroño, Paul Kluge, Sabine Kleinsteuber, Hauke Harms, Marcell Nikolausz

**Affiliations:** Department of Environmental Microbiology, Helmholtz Centre for Environmental Research–UFZ, 04318 Leipzig, Germany

**Keywords:** power-to-gas, renewable energy, biomethane, hydrogen, hydrogenotrophic methanogens, homoacetogenesis, methanogenic communities

## Abstract

The effects of the inoculum origin, temperature or operational changes on ex situ biomethanation by complex microbial communities have been investigated; however, it remains unclear how the diversity of the inoculum influences the process and its stability. We explored the effect of microbial diversity of four inocula (coded as PF, WW, S37 and Nrich) on methane production, process stability and the formation of volatile fatty acids as by-products. The highest methane amounts produced were 3.38 ± 0.37 mmol, 3.20 ± 0.07 mmol, 3.07 ± 0.27 mmol and 3.14 ± 0.06 mmol for PF, WW, S37 and Nrich, respectively. The highest acetate concentration was found in less diverse cultures (1679 mg L^−1^ and 1397 mg L^−1^ for S37 and Nrich, respectively), whereas the acetate concentrations remained below 30 mg L^−1^ in the more diverse cultures. The maximum concentration of propionate was observed in less diverse cultures (240 mg L^−1^ and 37 mg L^−1^ for S37 and Nrich cultures, respectively). The highly diverse cultures outperformed the medium and low diversity cultures in the long-term operation. Methanogenic communities were mainly composed of hydrogenotrophic methanogens in all cultures. Aceticlastic methanogenesis was only active in the highly diverse sludge community throughout the experiment. The more diverse the inocula, the more methane was produced and the less volatile fatty acids accumulated, which could be attributed to the high number of microbial functions working together to keep a stable and balanced process. It is concluded that the inoculum origin and its diversity are very important factors to consider when the biomethanation process is performed with complex microbial communities.

## 1. Introduction

Renewables such as photovoltaics and wind power may produce temporary surplus electricity that needs an energy storage solution. Power-to-gas (P2G) has gained attention to enable the storage of surplus electricity in the form of a storable gas such as hydrogen or methane [1]. Hydrogen can be used as a fuel and chemical feedstock or stored in the natural gas grid up to certain limits [2] according to country regulations. Methane, on the other hand, is more advantageous because it can be used in the same applications as hydrogen with the advantage of higher volumetric energy content [3]. Methane may be a better option because it is more compatible with the existing gas infrastructure allowing direct grid injection and the higher energy density of methane makes its transport and storage easier [3]. However, CH_4_ is a more potent greenhouse gas than CO_2_; therefore, its leakage should be avoided during the process and distribution. Moreover, if CH_4_ is oxidized again to CO_2_ in an industrial setup, the resulting CO_2_ could be reconverted to CH_4_ with renewable hydrogen once again in a circular process.

Considering methane as the energy carrier of choice for the P2G concept, the first step of the process is hydrogen production through water electrolysis (Equation (1)) followed by a methanation step in which hydrogen drives the reduction of carbon dioxide to methane (Equation (2)) [4].

(1) $$4H_{2}O\;\;\to \;2O_{2}+4H_{2}$$

(2) $$4H_{2}+CO_{2}\to \;CH_{4}+2H_{2}O$$

The methanation step is either a catalyst-based chemical reaction (Sabatier process) or a biochemical reaction (hydrogenotrophic methanogenesis). The biochemical reaction can be performed with pure cultures [5] or mixed cultures [6], and depending on the type of biocatalyst the bioprocess can be classified as in situ, ex situ or hybrid biomethanation as described in detail by [7]. Several studies reporting the use of mixed cultures in the aforementioned bioprocesses have been summarized in review papers [6,8,9].

Injecting hydrogen to reactors containing mixed cultures often leads to the accumulation of volatile fatty acids (VFA) such as acetate, propionate, butyrate or even longer-chain or branched C4 and C5 organic acids. This has been observed during in situ [10,11,12,13,14], ex situ [7,15,16,17,18] or hybrid biomethanation [19]. Additionally, it was recently shown that formate is also produced and consumed during H_2_/CO_2_ metabolism with mixed cultures [15,20]. The production of acetate or longer-chain organic acids is due to the enhancement of homoacetogenesis and/or chain elongation reactions. The produced acetate is converted to methane if aceticlastic methanogens are present in the mixed cultures [6]. However, acetate can be used to build microbial biomass as well. Another possibility for acetate consumption is mediated by syntrophic acetate-oxidizing bacteria (SAOB) that convert acetate into H_2_ and CO_2_ if the H_2_ partial pressure is low enough to make the reaction thermodynamically feasible [21].

Different aspects, such as reactor configuration [7], process operation mode [18,22,23,24], methods to improve gas delivery [25,26,27,28], temperature, inoculum [29], and pH [30,31], affect the process. Since temperature is a deterministic factor influencing the microbial community structure in anaerobic digestion [32,33,34] or methanogenic activity and diversity of methanogenic communities in natural environments [35], it has also been widely studied in engineered systems. Recent studies have investigated the effect of temperature in gas fermentation of H_2_/CO_2_ or syngas [18,31,36,37,38,39,40,41,42,43], and one study modeled syngas biomethanation under mesophilic and thermophilic conditions and found that thermophilic communities showed higher specific methane productivity (18.8 mmol/g VSS/d) than mesophilic counterparts and that modulating the partial pressure of CO_2_ can boost the product selectivity towards methane [44]. Another study found that psychrophilic conditions can inhibit methanogenic activity but either mesophilic or psychrophilic conditions can enrich homoacetogens [45]. To better understand the microbial communities and their functioning, omics techniques such as metagenomics and metatranscriptomics have been exploited to unravel in more detail the community members and their metabolic functions during biomethanation of hydrogen [46,47,48,49,50,51,52,53].

Recent biomethanation studies have identified the pathways under mesophilic and thermophilic conditions [54], combined experimental and model data to dissect the competition between methanogens and homoacetogens [55], determined how functional redundancy leads to quick recovery of the methane production rate [24], analyzed the carbon flow during methanogenesis inhibition [20], and revealed microbial community changes during syngas biomethanation in trickle bed reactors with different nutrient sources, including non-sterile digestates [56]. The enhancement of the process can be achieved by addition of zero valent iron (ZVI) [57], by introducing micro-porous materials as enhancer of biofilm immobilization and hydrogen mass transfer during ex situ biomethanation in trickle bed reactors [58] or via bioaugmentation with hydrogenotrophic methanogens (*Methanoculleus bourgensis* and *Methanothermobacter thermautotrophicus)* as active microbial resource management during mesophilic and thermophilic in situ biomethanation [59]. Moreover, the enrichment of hydrogenotrophic methanogens during in situ biomethanation [60] and the microbial successions and carbon flow were investigated in standard continuous stirred tank reactors [61].

A recent study indicated a link between the inoculum origin and the acetate consumption rate [10], while other studies have found that inoculum sources (mesophilic and thermophilic) or sludge inocula from different types of reactors [17,62] play an important role during biomethanation of hydrogen. However, it remains unclear how the diversity of the inoculum affects the biomethanation process. Hitherto, little attention has been given to the influence of the inoculum diversity on the stability and performance of the biomethanation process. This study fills this gap by testing cultures of high, medium and low diversity under similar operational conditions.

The present study compared inocula of different diversity regarding the process performance in biomethanation of hydrogen. It was hypothesized that the inoculum diversity influences the process, especially the unwanted accumulation of acetate. Process parameters were closely monitored and the microbial community composition was studied via terminal restriction fragment length polymorphism (T-RFLP) of *mcr*A (for methanogens) and 16S rRNA genes (for bacteria).

## 2. Materials and Methods

### 2.1. Experimental Setup

Four inocula were used to set up hydrogen biomethanation reactors operating in fed-batch mode. The first inoculum (termed WW) was anaerobic granular sludge sampled from an industrial-scale upflow anaerobic sludge blanket (UASB) reactor treating wastewater from paper industry. The second sludge inoculum (PF) originated from a pilot-scale plug flow reactor digesting cow manure and maize silage. WW and PF sludge inocula were degassed for 5 days at 37 °C before starting the experiment. The third inoculum (S37) was derived from an alkali-tolerant enrichment culture digesting wheat straw, which was established in a previous study in our laboratory [63]. The fourth inoculum (Nrich) originated from a regularly maintained hydrogenotrophic enrichment culture obtained in our previous study [15]. The rationale for using these cultures was (a) we have previously demonstrated that they were able to grow in the same medium and (b) the cultures already had a high, medium and low diversity in the inoculum used for the setup, which made them suitable to test our hypothesis.

A 500 mL master inoculum mixture was prepared for each inoculum by mixing 10% (*v*/*v*) inoculum with a mineral medium described in our previous study (Medium A; [15]) under anoxic conditions. The initial pH was set to 9.0 for every master inoculum with a sterile anoxic stock solution of 2 M KOH. Then, while stirring inside the anaerobic glove box, all reactor experiments were started in serum bottles of 219.5 mL by filling 50 mL of master inoculum mixture, sealing with butyl rubber stoppers and crimping with aluminum caps. Every master mixture was used to inoculate four biological replicates being fed with a gas mixture of H_2_/CO_2_ (4:1) and a total pressure of ~2.2 bar as described by [15]. Three biological replicates with N_2_/CO_2_ (4:1) in the headspace were set up with each inoculum to determine the background biogas production from the sludge or cell debris. The gas phase was replenished every 24 h, except on weekends. The reactors were incubated at 37 °C and shaken at 200 rpm. Medium was replenished on weekly basis by withdrawing five milliliters of broth and adding an equal volume of fresh medium. Measurements of the gas phase were performed at the end of each batch cycle, whereas the liquid phase was analyzed every 7 days. The gas amount is presented in mmol and was calculated as described by [15].

### 2.2. Analytical Methods

A high-resolution manometer (LEO 5, Keller, Switzerland) was used to measure the pressure drop. The gas composition was analyzed by gas chromatography (GC). Organic acids were measured by high performance liquid chromatography (HPLC) after sampling 1.5 mL of broth. Detailed information about pressure measurement, GC and HPLC setup, and sample preparation was given in our previous study [15]. Samples were stored at −20 °C if not measured immediately. The pH value was recorded 90 s after loading 200 µL of broth in a mini-pH meter (ISFET pH meter S2K922, ISFETCOM Co., Ltd., Hidaka, Japan). All process parameters were monitored at the beginning of the experiment and every seven days until day 49.

### 2.3. Microbial Community Analysis

We analyzed the microbial community sampled at the start of the experiment and then every seven days. The samples (1.5 mL) were centrifuged at 4 °C and 20,817× *g* for 10 min and the cell pellets stored at −20 °C until DNA extraction. DNA from the pellet was extracted with the NucleoSpin^®^Soil Kit (MACHEREY-NAGEL GmbH& Co. KG, Düren, Germany). The buffers SL2 and SX were used. Extracted DNA was stored at −20 °C until use. The microbial community structure was screened by T-RFLP analysis of *mcrA* genes for methanogens and 16S rRNA genes for bacteria.

PCR amplification for T-RFLP analysis of the methanogens was targeting the *mcrA* genes using forward primer mlas (5′-GGT GGT GTM GGD TTC ACM CAR TA-3′) and reverse primer mcrA-rev (5′-CGT TCA TBG CGT AGT TVG GRT AGT-3′, FAM labelled) as described by [64]. All primers were purchased from Eurofins Genomis Germany GmbH. Each reaction contained the following reactants: 6.25 µL MyTaq 2× Mastermix (Bioline GmbH, Luckenwalde, Germany), 5 pmol of each primer, 3.85 µL PCR grade water and 1 µL of extracted DNA (containing on average 40–50 ng of DNA). The final volume was 12.5 µL. The PCR started with a denaturation step at 95 °C for 3 min, followed by 5 cycles of 20 s at 95 °C, 20 s at 48 °C and 15 s at 72 °C with an increase in temperature of 0.1K/s, followed by 25 cycles of 20 s at 95 °C, 20 s at 55 °C and 15 s at 72 °C. For the final elongation, the temperature was 72 °C for 10 min.

For 16S rRNA genes, the primers UniBac27f (5′ GAG TTT GAT CMT GGY TCA G 3′, FAM labelled) and UniV1492r (5′ TAC GGY TAC CTT GTT ACG ACT T 3′) were used. The PCR reaction mixture and thermocycling protocol was carried out as described by [65].

Amplicons were purified as previously described by [66]. T-RFLP analysis of purified PCR products was carried out after digestion with the restriction enzymes *Bst*NI (for *mcr*A amplicons) and *Rsa*I (for 16S rRNA amplicons) using the sequencer ABI PRISM 3130xl Genetic Analyzer (Applied Biosystems, Foster City, CA, USA). Resulting electropherograms were processed as described by [67]. During analysis, signals with low peak areas were removed by using cutoffs 7 (*mcrA*) and 4 (16S rRNA) times the standard deviation of the datasets. Terminal restriction fragments (T-RFs) with abundances of less than 1% were filtered out and the sum of the remaining T-RFs was set to 100%. The *mcrA*-derived T-RFs were taxonomically assigned by using a T-RF database generated in our laboratory [68]. The 16 S rRNA-derived T-RFs were not taxonomically assigned and each fragment size is shown in the bar charts. T-RFLP data were used to calculate the ecological indices quantifying the α-diversity of bacterial and methanogenic communities as described by [67]. This paper presents the richness (R), diversity of order one (^1^D), diversity of order two (^2^D) and evenness of order one (^1^E).

### 2.4. Statistical Analysis

Statistical analysis (comparison of process performance parameters or ecological indices between different sampling days) was performed as detailed in our previous study [62]. Analysis of variance (ANOVA) was used to find out if there are significant differences. For multiple comparisons between sampling days, we used Tukey’s post-hoc test in order to identify where the differences are.

## 3. Results

Four inoculum sources with different microbial diversity were used for fed-batch biomethanation of hydrogen. During the experiment, the process parameters and microbial community structure and diversity were investigated.

### 3.1. Process Performance

**Figure 1:**
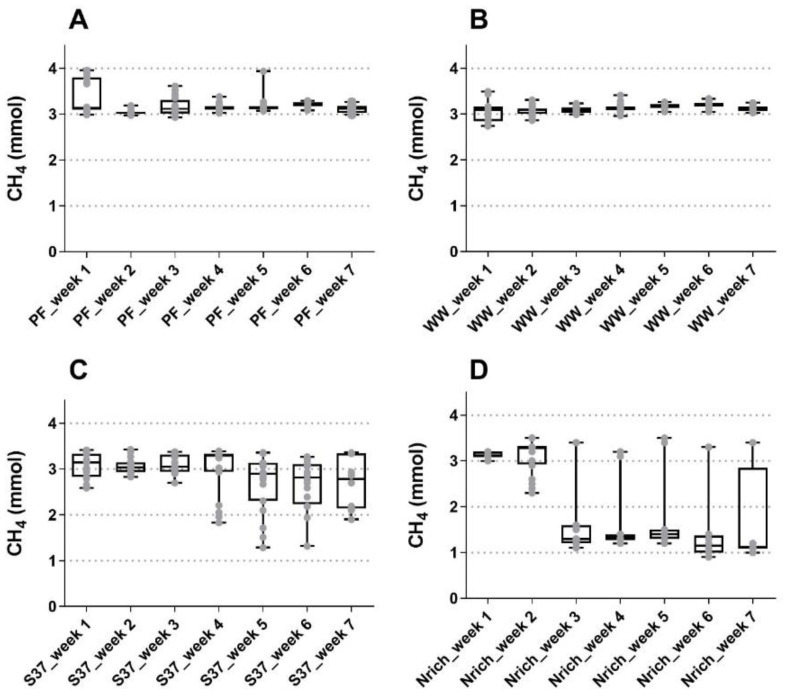
Boxplot showing the produced methane of samples taken for 5 days a week for all biological replicates combined. The reactors were fed every 24 h (except weekends) and measurements were performed at the end of each batch cycle. (**A**) Reactors PF (inoculated with digestate from a pilot-scale plug flow reactor digesting cow manure and corn silage); (**B**) reactors WW (inoculated with anaerobic granular sludge from an industrial-scale UASB reactor treating wastewater from paper industry); (**C**) reactors S37 (inoculated with an enrichment culture digesting wheat straw); (**D**) reactors Nrich (inoculated with a hydrogenotrophic enrichment culture). Horizontal bars depict the mean and whiskers represent the range of all values. Values for all four replicates per week are shown with grey filled circles.

The gas phase was monitored on daily basis except weekends and the performance regarding methanogenesis was summarized in Figure 1. Methane amounts were quite similar during the first three weeks of fed-batch cultivation for all inocula, although the Nrich cultures produced approximately 45% less methane than the other cultures in the third week. The highest methane amounts were 3.38 ± 0.37 mmol, 3.07 ± 0.27 mmol and 3.14 ± 0.06 mmol for PF, S37 and Nrich, respectively, in week 1, and 3.20 ± 0.07 mmol for WW in week 6. Statistical comparison between all seven weeks showed no significant differences in the methane amounts for PF, WW and S37. This observation is consistent with the results shown in Figure 1A–C, which indicate a very stable process performance for both sludge inocula whereas the S37 cultures showed a rather variable methane production across replicates in the last four weeks. The situation was different for the Nrich inoculum because the mean values of methane produced from week 3 to 7 were all significantly lower than those of week 1 and 2. The atypical maximum values from week 3 to 7 correspond to batch cycles with a fermentation time of 72 h for all biological replicates (Figure 1D). The hydrogenotrophic community Nrich showed a remarkably lower performance than PF and WW despite being highly enriched in hydrogenotrophic methanogens. The average methane amounts produced during 49 batch cycles were 3.18 ± 0.20 mmol, 3.12 ± 0.12 mmol, 2.92 ± 0.47 mmol and 2.11 ± 0.99 mmol for PF, WW, S37 and Nrich, respectively.

### 3.2. Organic Acids Profiles

**Figure 2:**
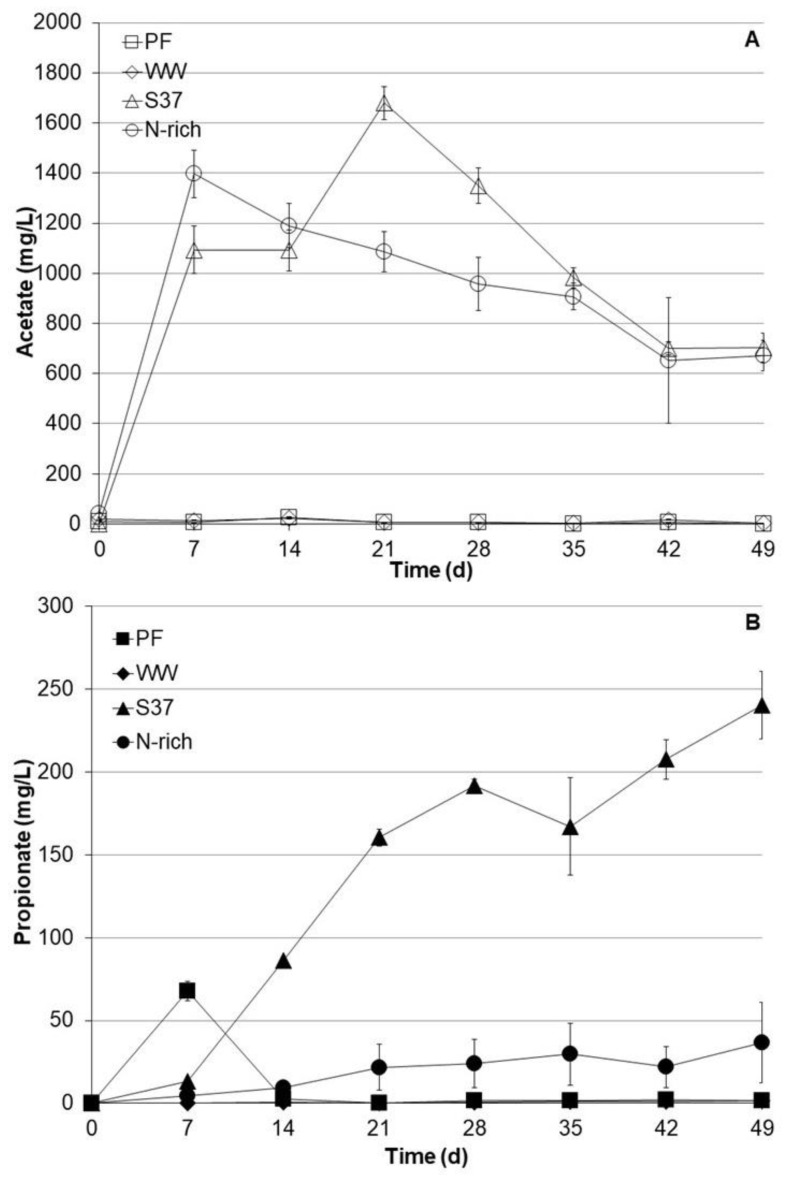
Profiles of acetate (**A**) and propionate (**B**) concentrations during autotrophic cultivation with H_2_/CO_2_ (80:20). Reactors PF (inoculated with digestate from a pilot-scale plug flow reactor digesting cow manure and corn silage); reactors WW (inoculated with anaerobic granular sludge from an industrial-scale UASB reactor treating wastewater from paper industry); reactors S37 (inoculated with an enrichment culture digesting wheat straw); reactors Nrich (inoculated with a hydrogenotrophic enrichment culture). Error bars represent the standard deviation of the mean of *n* = 4.

VFA such as acetate and propionate were monitored on weekly basis (Figure 2). The S37 and Nrich cultures produced substantial amounts of acetate. The acetate concentrations after 7 days were 1094 ± 145 mg L^−1^ and 1397 ± 94 mg L^−1^ for S37 and Nrich, respectively. The peak acetate concentration for S37 was 1679 ± 697 mg L^−1^ on day 21. Acetate concentrations started to decrease after 7 and 21 days, reaching 703 ± 58 mg L^−1^ and 672 ± 62 mg L^−1^ after 49 days for the S37 and Nrich cultures, respectively. In contrast, the acetate concentrations in the PF and WW cultures were negligible (below 30 mg L^−1^) throughout the experimental period. Propionate profiles were distinct for each inoculum. In PF cultures, propionate peaked at 68 ± 6.0 mg L^−1^ after 7 days but dropped to below the detection limit after 14 days. Propionate concentrations in WW cultures were below the detection limit throughout the experiment. The situation was different for S37 and Nrich since both started to accumulate propionate but to different extents. S37 produced the most propionate with a maximum concentration of 240 ± 20 mg L^−1^ after 49 days, whereas Nrich cultures contained 37 ± 4.2 mg L^−1^ at the end of the experiment. Figure 2 clearly shows that S37 produced significantly higher amounts of propionate than Nrich, PF and WW.

### 3.3. pH Profiles during Autotrophic Cultivation

**Figure 3:**
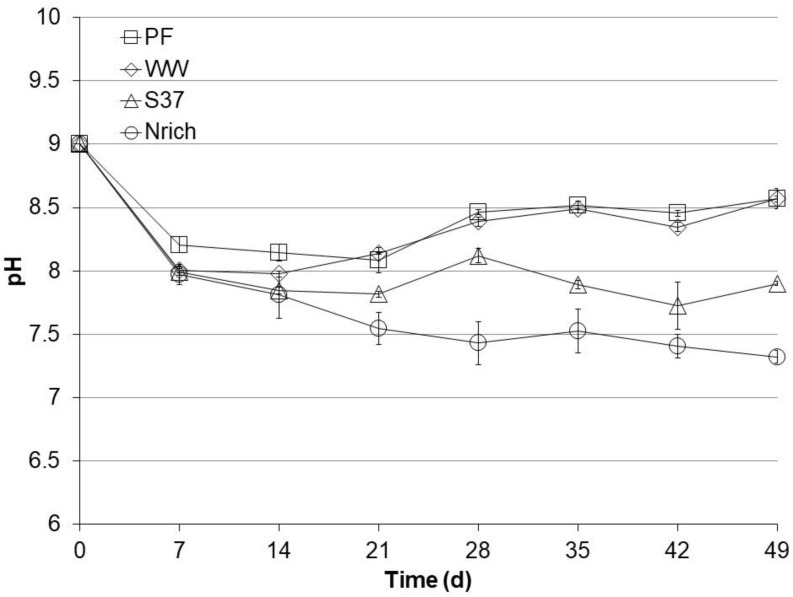
pH profiles during autotrophic cultivation with H_2_/CO_2_ (80:20). Reactors PF (inoculated with digestate from a pilot-scale plug flow reactor digesting cow manure and corn silage); reactors WW (inoculated with anaerobic granular sludge from an industrial-scale UASB reactor treating wastewater from paper industry); reactors S37 (inoculated with an enrichment culture digesting wheat straw); reactors Nrich (inoculated with a hydrogenotrophic enrichment culture). Error bars represent the standard deviation of the mean of *n* = 4.

The pH of the medium was set to 9 before starting all cultures. After 49 days, the cultures could be divided in two groups according to their final pH values (Figure 3). The pH of each sampling day was statistically compared among the tested inocula because pH changes can alter the ecophysiology of microbial communities and therefore the performance. There were no significant pH differences between PF and WW during the entire experiment. The pH values of the PF and WW cultures were significantly higher than those of the S37 and Nrich cultures after 14, 21, 28, 35 and 49 days. PF and WW had a high pH of 8.6 ± 0.001, S37 had an intermediate pH of 7.9 ± 0.02 and Nrich had a low pH of 7.3 ± 0.05. The higher pH of PF and WW corresponded to lower concentrations of acetate and propionate (Figure 2) than in S37 and Nrich cultures of lower pH values.

### 3.4. Changes in Diversity of Microbial Communities

**Figure 4:**
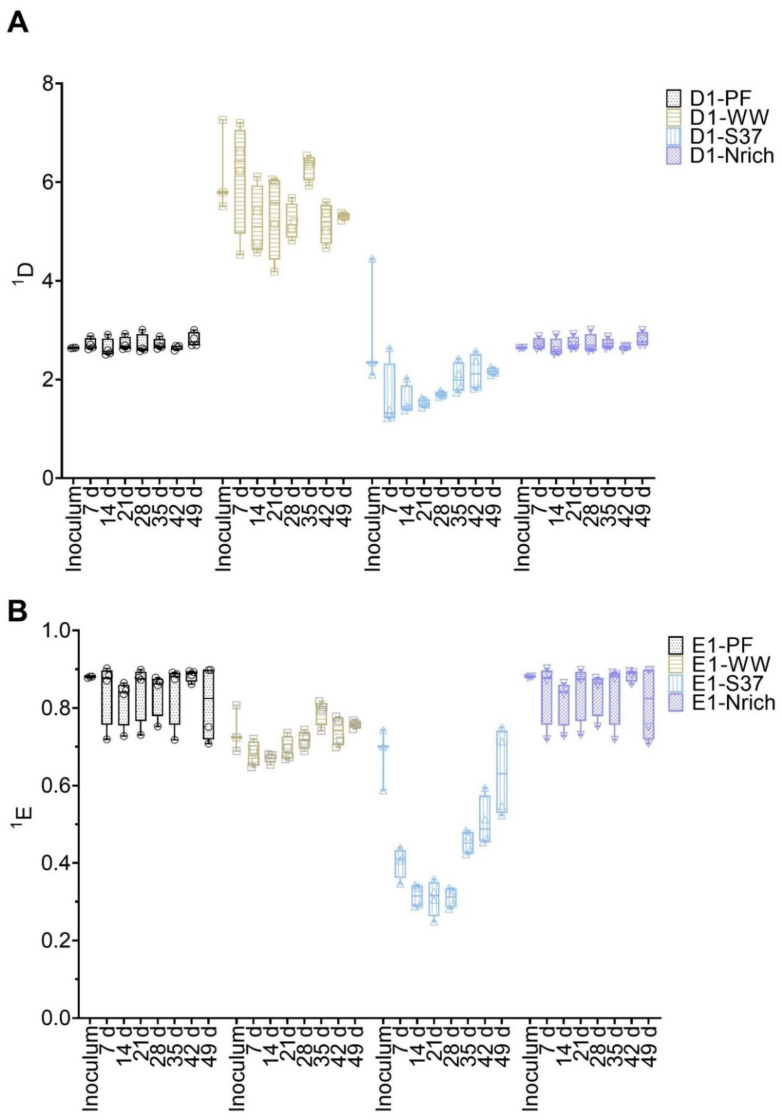
Box plots of diversity (**A**) and evenness (**B**) for q = 1 at different sampling times for the methanogenic communities. Note that for inoculum, *n* = 3, whereas *n* = 4 for all remaining sampling days. Inoculum descriptions as previously described in Figure 1.

Diversity and evenness of order one were calculated from T-RFLP data to assess the α-diversity of bacterial and methanogenic communities (Figure 4). ANOVA analysis of both indices resulted in significant differences for methanogens as well as for bacteria. To find out the differences between cultures derived from various inocula, we used Tukey’s post-hoc test.

The methanogenic community of the WW inoculum was significantly more diverse than those of PF, S37 and Nrich (*p* < 0.0001) (Figure 4A). The diversity indices of the methanogenic communities of the inocula PF, S37 and Nrich were not statistically different (Figure 4A). ^1^D of the methanogenic community did not differ between the sampling days for both PF and WW. More importantly, ^1^D of the methanogenic community of WW was significantly higher than those of PF, S37 and Nrich on each sampling day (*p* < 0.0001). Afterwards, a comparison of evenness ^1^E was performed. The methanogenic community of the Nrich inoculum was significantly more even than that of S37, and the result was the same when comparing PF to S37 (*p* = 0.02 for both comparisons) (Figure 4B). Comparing PF to WW on each sampling day showed no differences in ^1^E. The methanogenic community of PF was significantly more even than that of S37 on each sampling day (*p* < 0.0001 for days 7, 14, 21, 28, 35 and 42, and *p* = 0.01 for day 49). A similar result was observed when comparing WW to S37 (*p* < 0.0001 for days 7, 14, 21, 28, 35 and *p* = 0.0001 for day 42), except for day 49 that showed no differences.

**Figure 5:**
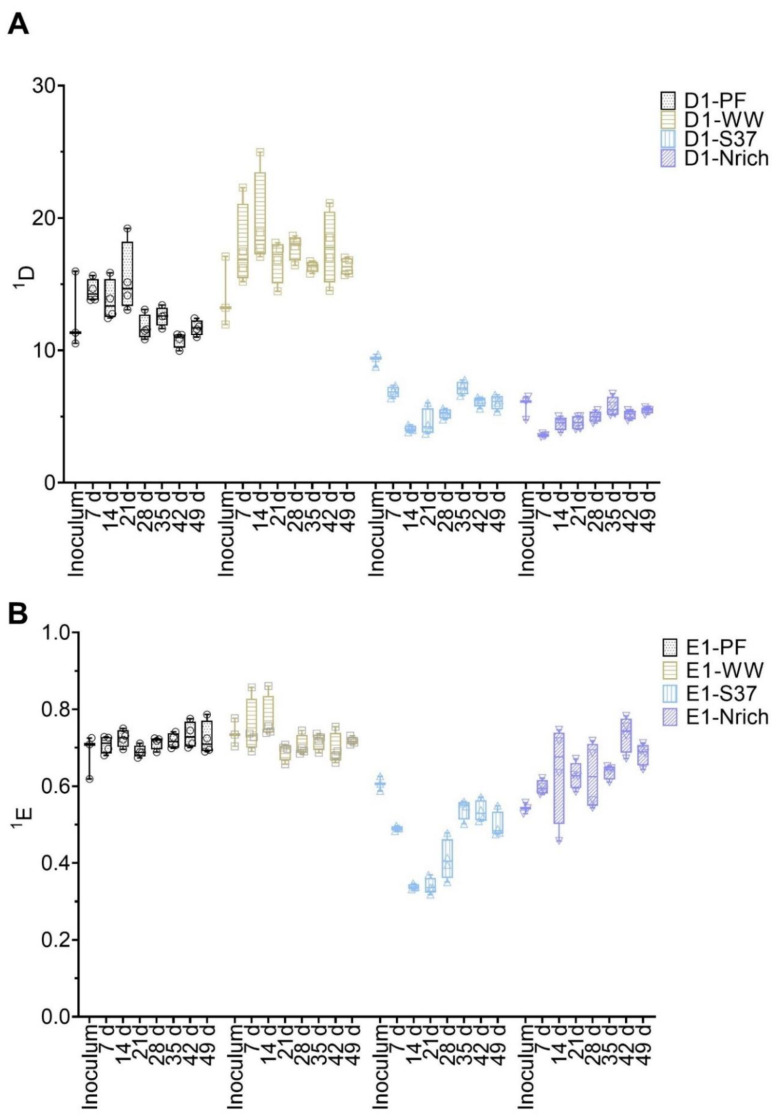
Box plots of diversity (**A**) and evenness (**B**) for q = 1 at different sampling times for the bacterial communities. Note that for inoculum, *n* = 3, whereas *n* = 4 for all remaining sampling days. Inoculum descriptions as previously described in Figure 1.

The bacterial communities’ diversity and evenness are shown in Figure 5. First, the differences between inoculum samples were evaluated. There were no significant differences between the inocula of the following: PF vs. WW, PF vs. S37 and S37 vs. Nrich. However, both PF and WW had a significantly higher ^1^D compared to Nrich (*p* < 0.0001 for both comparisons) but only WW was superior to S37 (*p* = 0.02). Taking into consideration one inoculum at the time and comparing ^1^D on each sampling day showed no significant differences for most inocula. The only exception was PF because ^1^D on day 21 was higher than on day 42 (*p* = 0.01). Next, we compared ^1^D between the cultures on each sampling day. After 7 days, the cultures PF and WW were significantly more diverse than S37 and Nrich (*p* < 0.0001 for both comparisons). On day 14, WW was significantly more diverse than PF and ^1^D of PF and WW were higher than those of S37 and Nrich (*p* < 0.0001 for both comparisons). ^1^D was significantly higher for the following comparisons: PF vs. S37 (day 21, 28, 35, 42 and 49), PF vs. Nrich (day 21, 28, 35, 42 and 49), WW vs. S37 (day 21, 28, 35, 42 and 49) and WW vs. PF (day 14, 28, 42 and 49). However, there were no differences between WW and PF on days 21 and 35. Afterwards, a comparison of ^1^E was performed. Only the inoculum comparison PF vs. Nrich (*p* = 0.03) and WW vs. Nrich (*p* < 0.0001) showed a significantly higher value in ^1^E. ^1^E of PF and WW were significantly higher than that of S37 (comparisons for all sampling days). Lastly, WW had a significantly higher value in ^1^E compared to Nrich but only on days 7 and 14.

### 3.5. Relationship between the Richness of Methanogenic Communities and Methane Production

**Figure 6:**
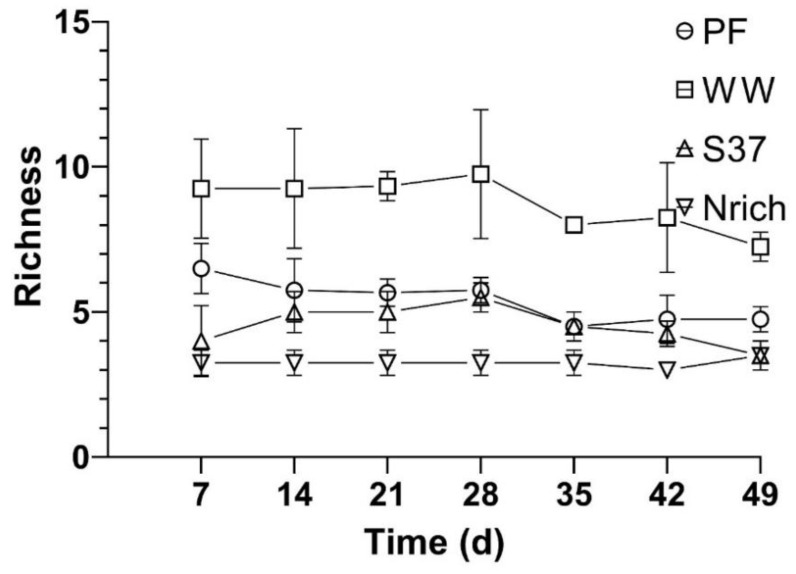
Richness of the methanogenic communities derived from T-RFLP analysis of the *mcr*A genes. Richness was calculated as the number of T-RFs with an abundance of >1%. The mean and standard deviation of *n* = 4 is shown.

To assess whether diversity had an effect on the methane amount that was produced by the different cultures we assembled four cultures with different inocula under identical conditions and monitored the methane production and methanogenic community. The richness of the methanogenic communities is shown every 7 days so that the changes in this ecological index upon H_2_/CO_2_ metabolism could be observed (Figure 6). Ranking the inocula from the largest to the smallest richness of methanogens for each sampling day showed a consistent behavior as follows: WW > PF > S37 > Nrich (Figure 6).

Furthermore, the results from the ANOVA and Tukey’s test (α = 0.05) revealed significant differences between the inocula for all sampling days. WW had a significantly higher richness than PF, S37 and Nrich for all sampling days. PF had significantly higher richness than S37 on days 7 and 49. The only two sampling times without significant differences between PF and Nrich were days 14 and 42. A comparison between S37 and Nrich showed significantly higher differences only on days 21 and 35.

**Figure 7:**
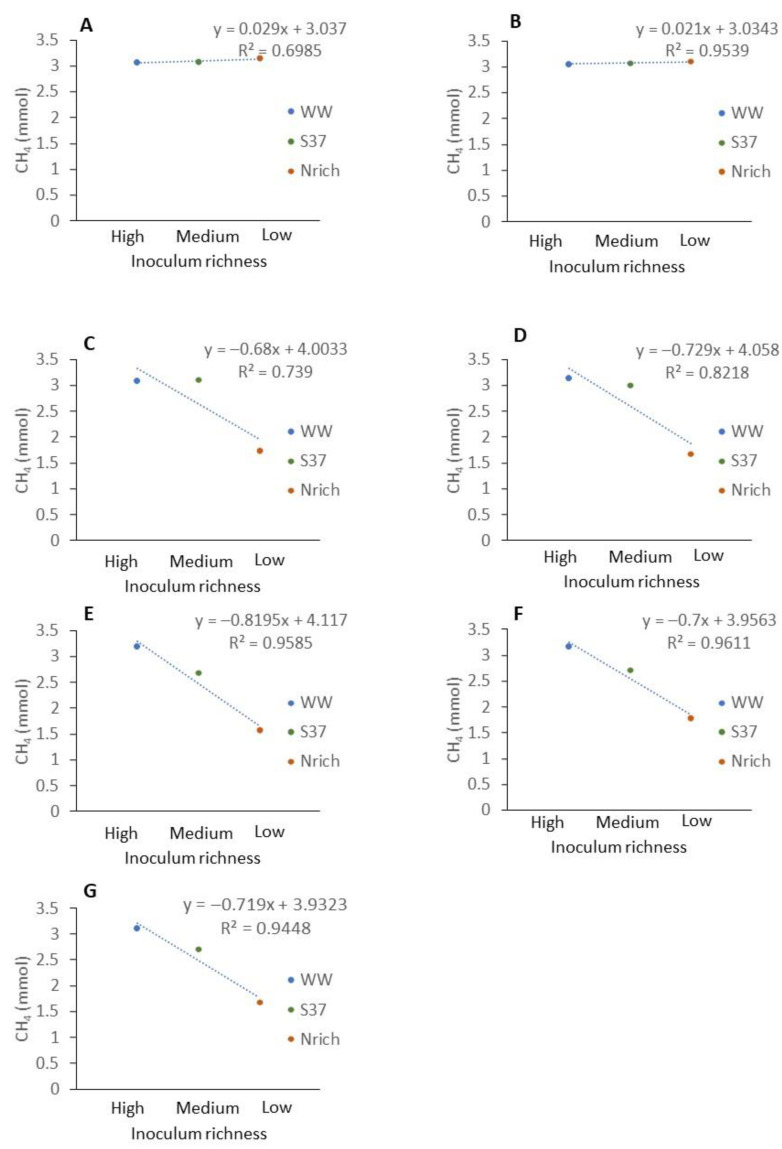
Relationship between the richness of methanogenic communities of different inocula and the methane amount produced at different sampling times. Description of the inocula as described in Figure 1. The mean values of *n* = 4 are shown. (**A**) 7 d, (**B**) 14 d, (**C**) 21 d, (**D**) 28 d, (**E**) 35 d, (**F**) 42 d and (**G**) 49 d.

The average methane amount produced by WW and PF was higher than that produced by S37 and Nrich (Figure 1). To analyze the relationship between the richness of methanogens and the amount of methane produced, we ranked the inoculum richness in the x axis from the highest to the lowest richness as follows: high (WW), medium (S37) and low (Nrich) richness. PF fell to a richness level similar to that of S37 (Figure 6). Plotting the classified inoculum type as a categoric variable and the methane amount as a numeric variable showed a decreasing trend for most sampling days except on days 7 and 14 (Figure 7A,B). The methane amount decreased proportionally with the richness of methanogens of the inocula (Figure 7C–G).

### 3.6. Changes in the Structure of the Microbial Communities

**Figure 8:**
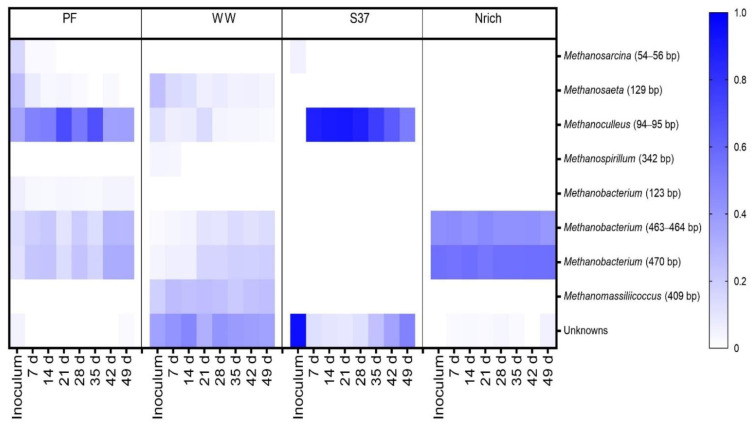
The composition of the methanogenic communities is shown, based on relative abundances of individual families. *mcrA*-derived T-RFs were taxonomically assigned using the T-RFLP database for methanogens in AD [69]. The restriction enzyme used was *Bst*NI. Note that for inoculum, *n* = 3, whereas *n* = 4 for all remaining sampling days. Inoculum descriptions are the same as described in Figure 1. T-RFs that could not be assigned to any database entry were grouped together as “Unknowns”. The T-RF of 470 bp may also correspond to *Methanomassiliicoccaceae*, but our previous study clarified that in our system it only corresponds to *Methanobacterium* [15].

T-RFLP fingerprinting coupled with a sequence database to assign taxonomy to T-RFs [68] was used to gain insights into the methanogenic communities. To visualize the families that were present or absent in different inocula and derived cultures, we plotted all families in a single heat map. The methanogenic community structure was distinct for each inoculum (Figure 8). Since more than one T-RF might be affiliated to the same methanogenic family or one T-RF could represent more than one family, the lengths of the T-RFs (in base) are also given in brackets after the taxa names. The inoculum of PF was dominated by hydrogenotrophic methanogens affiliated to the family *Methanoculleus* (94–95 bp). WW and S37 presented a high share of unknown T-RFs in the inoculum. *Methanobacterium* (470 bp) made more than 50% of the methanogenic community in the inoculum of Nrich. The T-RF of 470 bp length may also represent *Methanomassiliicoccus*; however, in our previous study, we discerned that in our system it only corresponded to *Methanobacterium* based on *mcr*A gene amplicon sequencing analysis [15].

T-RFs affiliated to *Methanosarcina* (54–56 bp) were present in the PF and S37 cultures only. Strict acetoclastic methanogens affiliated to *Methanosaeta* (129 bp) were the second most abundant taxon in the inoculum of WW with a relative abundance of 24%.

Different hydrogenotrophic methanogens became abundant in each community as the experiment proceeded. *Methanoculleus* (94–95 bp) dominated in PF throughout the experiment (35–70% relative abundance). In WW, unknown T-RFs ranged from 30% to 47%. *Methanoculleus* (94–95 bp) dominated the methanogenic community of the culture S37 (50–90% relative abundance), whereas *Methanobacterium* (470 bp) dominated in the Nrich culture (53–56% relative abundance). The T-RF *Methanobacterium* (470 bp) was present in PF, WW and Nrich, but not in S37. Likewise, *Methanoculleus* (94–95 bp) was present in S37, PF and WW, but not in Nrich. *Methanobacterium* (463–464 bp) was present in Nrich, PF and Nrich, but absent in S37. *Methanobacterium* (123 bp) was unique in PF, whereas *Methanospirillum* (342 bp) was only present in WW. *Methanosaeta* (129 bp) was present in PF and WW, which indicates that acetoclastic methanogenesis was functioning in both. Interestingly, *Methanomassiliicoccus* (409 bp) was only present in WW with a relative abundance ranging from 18% to 26% depending on the sampling day.

**Figure 9:**
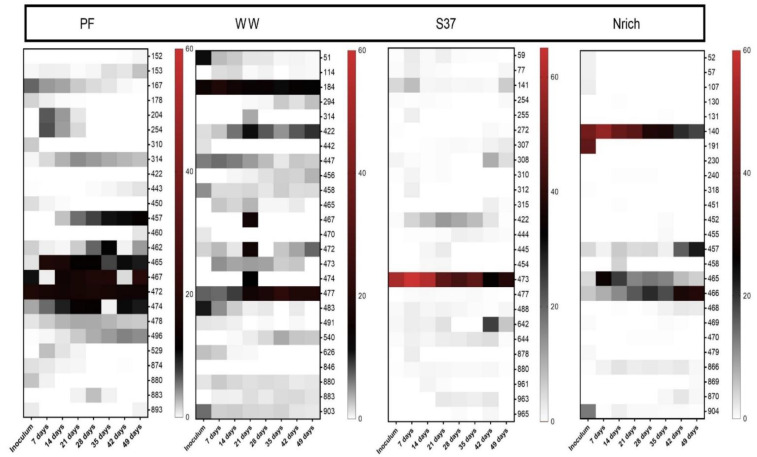
Composition of the bacterial communities based on relative T-RF abundance. The top 25 T-RFs based on 16S rRNA genes are shown. The restriction enzyme used was *Rsa*I. Note that for inoculum, *n* = 3, whereas *n* = 4 for all remaining sampling days. Inoculum descriptions are the same as described in Figure 1.

T-RFLP data from the bacterial communities did not allow taxonomic assignment due to a missing sequence database; thus, we focused on the 25 most dominant T-RFs. Each inoculum presented a different behavior as the relative abundance of some T-RFs increased, decreased or remained unchanged during the experimental time considering the initial relative abundance in the inoculum as the reference point (Figure 9).

## 4. Discussion

Although it is well known that inoculation of biomethanation reactors plays an important role in process performance and stability, the particular role of microbial diversity of the inoculum has not been thoroughly studied. To clarify the answer to this question, we selected two sludge inocula of different origin and two enrichment cultures generated in our laboratory. One enrichment culture was specialized in straw degradation [63] and the second one was hydrogenotrophic enrichment culture [15]. To exclude the effect of temperature as a driver of process performance change, we selected inocula that originated from mesophilic conditions only. In the beginning of the experiment, the methane amount was comparable for all inocula. However, the methane produced in batch cycles of 24 h decreased dramatically in the cultures inoculated with the enrichment cultures. In contrast, the methane amounts produced by the cultures inoculated with sludge remained in a similar range throughout the entire experiment. In fact, the methane amounts produced initially were similar to the values found in our previous studies [15,62], which indicates that the process results were comparable. Furthermore, the methane concentration of ≥90% was in the range of values found in the literature [6,7,26,69]. It was unexpected that the most diverse inocula outperformed an already accommodated hydrogenotrophic enrichment culture. Based on our observations it can be stated that the diversity of the inoculum played an important role in maintaining a high performing and stable process. Therefore, it is an important factor to consider for practical applications, especially when mixed cultures from biogas plants or wastewater treatment plants are available to perform biomethanation. Hydrogenotrophic enrichment cultures suffering from low production of methane and VFA accumulation can be remediated by applying microbial resource management measures such as refining the growth medium and using sodium sulfide as reducing agent as described recently in our study [15]. Furthermore, bioaugmentation with a diverse methanogenic community (e.g., sludge/anaerobic granules from WWTP) could be a simple and practical microbial resource management measure.

Acetate production and accumulation during biomethanation of hydrogen can be problematic if homoacetogenesis is stimulated and acetoclastic methanogenesis is not functioning. Previous studies have found acetate as a major by-product during biomethanation of hydrogen [7,11,12,26,29,70,71], whereas other studies have also found propionate, iso-butyrate and n-butyrate [13,18]. The occurrence of these by-products can be explained by the activity of homoacetogenic bacteria as they can produce acetate and small amounts of butyrate [72]. Propionate could be produced from amino acids during the recycling of biomass and its accumulation can be explained by the inhibition of propionate degraders due to high hydrogen partial pressure. The production of organic acids of higher carbon chain length can be also explained by chain elongation of acetate, which is enhanced by feeding hydrogen [73,74]. In this study, the cultures S37 and Nrich produced significantly more acetate than PF and WW. For S37 and Nrich cultures, this observation could be explained by (i) the high hydrogen partial pressure, which created a selective advantage for acetate production, (ii) the inhibition of acetate and propionate metabolizing syntrophic bacteria or low relative abundance of this functional group, (iii) the absence of acetoclastic methanogens and (iv) the lower diversity of S37 and Nrich communities compared with their WW and PF.

A previous study confirmed that the inoculum and the predominant methanogens are important for the performance of biomethanation [62]. We found that in enrichment cultures, only one methanogen was the most dominant (S37: *Methanoculleus*, Nrich: *Methanobacterium*), while the sludge derived cultures were more diverse. Our results are consistent with our previous results and the reports found in literature [7,15,29,47,62]. Thus, it can be inferred that these types of methanogens have a selective advantage when hydrogen is highly available. In the light of the aforementioned results, it seems that both *Methanoculleus* and *Methanobacterium* are needed for the most efficient process (WW and PF), while only one of them (as in S37 and Nrich) is not sufficient.

Diversity analyses based on ecological indices have been poorly reported in biomethanation of hydrogen research. One study quantified the α-diversity as means to ensure that the sequencing depth was sufficient to cover the microbial richness [7]. The same authors highlighted that the community complexity in serial upflow and bubble column reactors showed distinct ordination patterns between the different reactor types based on PCoA analysis [7]. Another study quantified the α-diversity based on amplicon sequencing data and found higher diversity under mesophilic conditions than under thermophilic conditions [75]. A similar finding was described in a recent publication but ecological indices (α- or β-diversity) were not calculated [29]. Hydrogen addition to reactors acts as a strong selection factor favoring hydrogenotrophic metabolism, resulting in a decrease in the diversity as reported previously [15,75]. In the current study, we observed that the richness of the methanogenic communities decreased after long-term feeding of hydrogen. We used only mesophilic inocula and the highest richness was found in the sludge inocula (PF and WW). This was expected, since the other two inocula (S37 and Nrich) originated from enriched communities utilizing a single substrate—either wheat straw (S337) or H_2_/CO_2_ (Nrich).

Figeac and colleagues highlighted that high microbial diversity enabled faster adaptation to changes in temperature during biomethanation of hydrogen [29]. Therefore, it can be inferred that diversity plays an important role in adaptation to new conditions. In the current study, the most diverse cultures produced higher amounts of methane than the less diverse ones, a finding supported by the linear relationship between the richness of the inocula and the amount of methane produced, especially in long term operation (Figure 6).

The advantages of mixed cultures such as sludge are: (i) their complex microbial communities harboring several metabolic functions and allowing stable process performance; (ii) the ability to withstand starvation periods, which can be explained by the dominant methanogens present in the inoculum; (iii) functional redundancy as recently demonstrated by [24,62]; and (iv) fewer concerns with regard to the purity of the input gas. To explore our findings in more detail, further investigations with amplicon sequencing and metagenomics are needed to identify the taxonomy and potential metabolic functions of the microbial communities.

## 5. Conclusions

This study showed that microbial diversity is an important parameter to consider when performing biomethanation with mixed cultures. We report that a complex inoculum such as sludge from a wastewater treatment plant is suitable for biomethanation of hydrogen based on the observation of stable process performance and low amounts of by-products. The potential benefits of using sludge biomass to perform biomethanation also offer the possibility to withstand idle operation periods created by the intermittent nature of renewable energy. Additionally, the availability of large amounts of microbial biocatalyst in industrial scale reactors makes anaerobic sludge from wastewater treatment plants suitable for further exploration of the advantages and pitfalls of large-scale trials. This would reduce the risk in the technological advancement of biological P2G so that the renewable energy sector could be coupled to the wastewater treatment sector. Future research may explore the potential benefit of existing processes from bioaugmentation and re-inoculation strategies to increase the microbial diversity and functional redundancy in order to improve the biomethanation process.

## Data Availability

The data presented in this study are available in this article.
